# Research on Localization Algorithms Based on Acoustic Communication for Underwater Sensor Networks

**DOI:** 10.3390/s18010067

**Published:** 2017-12-28

**Authors:** Junhai Luo, Liying Fan, Shan Wu, Xueting Yan

**Affiliations:** School of Electronic Engineering, University of Electronic Science and Technology of China, Chengdu 611731, China; liy_fan@std.uestc.edu.cn (L.F.); wushansr33@gmail.com (S.W.); yxte_mail@163.com (X.Y.)

**Keywords:** acoustic communication localization, underwater sensor network, survey, spatial coverage, range measurement

## Abstract

The water source, as a significant body of the earth, with a high value, serves as a hot topic to study Underwater Sensor Networks (UWSNs). Various applications can be realized based on UWSNs. Our paper mainly concentrates on the localization algorithms based on the acoustic communication for UWSNs. An in-depth survey of localization algorithms is provided for UWSNs. We first introduce the acoustic communication, network architecture, and routing technique in UWSNs. The localization algorithms are classified into five aspects, namely, computation algorithm, spatial coverage, range measurement, the state of the nodes and communication between nodes that are different from all other survey papers. Moreover, we collect a lot of pioneering papers, and a comprehensive comparison is made. In addition, some challenges and open issues are raised in our paper.

## 1. Introduction

The water covers over 70% of the earth while just a small part of it has been discovered. As a significant body of the land, the water contributes to the transportation, nourishment and natural resources, etc. Therefore, how to make use of water resources becomes a hot topic, and plenty of research results have been reported. Underwater Sensor Networks (UWSNs), which can be used for underwater explorations, are a valuable research direction for realizing the underwater applications. UWSNs consist of a number of autonomous and individual sensor nodes [1]. Those sensor nodes are spatially distributed in underwater and carry out some sensing work aiming at obtaining water-related properties [2]. Various applications handle sensed data based on UWSNs with different demands. The state of the sensor node can be stationary, mobile or hybrid, which can transmit the information by the wireless connectivity. Meanwhile, Wireless Sensor Networks (WSNs) and many efficient technologies have been presented such as GPS, Radio Frequency (RF), and Visible Light Communication (VLC) for the terrestrial positioning. However, the frequencies of RF signals that are suitable for use in UWSNs are only 30–300 Hz. Meanwhile, there is a requirement that the antenna is large or the transmission power is high. The optical signals also suffer from the attenuation and scattering in the underwater. Hence, both of the technologies are not suitable for UWSNs. Fortunately, acoustic waves frequency is low lying between 10 Hz and 1 MHz [1], which can offer small bandwidth but has long wavelengths. It means that the acoustics can be used for relaying information over kilometers [3]. Acoustic communication establishes the links by using the propagation of the sound waves, which still suffer some challenges. In our paper, we will introduce localization algorithms based on acoustic communication for underwater sensor networks.

Thanks to the limitations of the computational power, the memory, the cost, the communication range and the lifetime of an individual sensor, the deployment of UWSNs is still a challenging job [1]. How many sensor nodes are arranged is a problem to which we should pay attention. A greater number of sensor nodes means a larger coverage range and higher precision but may increase the cost. As we know, the power of the sensor node is limited, which means that an effective strategy should be proposed to the system performance without shortening the operating time. In [1], the authors describe the difference between UWSNs and terrestrial sensor networks in three aspects, namely, dynamics, being error-prone and 3D. In UWSNs, the sensor node can move with the ocean currents, which lead to the property of dynamics. In addition, numerous factors such as water temperature, signal attenuation, dynamics, and noise may affect the performance of the sensor network, which results in the highly sensitive property. In addition, the random deployment of nodes forms the 3D architecture underwater.

In the terrestrial WSN, localization is a critical application and plenty of research results have been published. However, as we presented before, those types of signals (RF, optical signal) suffer the attenuation in the underwater. Underwater, the nodes can be divided into two types: the anchor node for which the position is known and the unknown node for which the position needs to be obtained by the localization algorithm [4]. Many types of methods can obtain the positioning information of the anchors. There we list two of them. First, the anchor node can be fixed at the known position, which means that the coordinates of the anchors are pre-configured. Second, the anchors can use specialized hardware to obtain their location such as GPS. In general, positioning algorithms use the distance or the angle information to localize the unknown node. The localization techniques can be classified into angulation and lateration [4]. The angulation uses the angle information while the literature uses the distance information. Moreover, some localization schemes do not need the anchor nodes to help realize positioning. In [5,6], the authors utilize the connectivity information to estimate the unknown node.

Currently, researchers have proposed many localization algorithms for UWSNs. However, most of the survey papers do not focus on the positioning algorithms. In [7], the authors present the survey of routing protocols in UWSNs. A paper [8] collects the recent advances on UWSNs. Some researchers consider the security and privacy in the localization of UWSNs [5]. In [9], the application, advances, and challenges are presented. Of course, some papers focus on the positioning algorithms. The papers [4,10,11] classify the localization algorithms based on the distributed vs. centralized, and estimation vs. prediction. In fact, the papers [10,11] just describe only the two types of algorithms and compare with a few type of aspects of the papers. The paper [4] is more detailed than [10,11] and collects more than twenty types of algorithms. However, it is not enough. In [12], the authors collect the range-based algorithms and compare the features of the algorithms. However, the less than thirty algorithms collected and compared do not contain most of the localization algorithms. Then, in [13], the authors classify the localization algorithms according to the mobility of the sensor nodes. Compared with other survey papers, the paper collects more research results; however, the other UWSN related information is not informed. Only the papers published before 2012 are collected, and some new research results have been reported that are not included. Thus, it is necessary to do a new survey about the localization algorithms.

Our paper collects the papers in this field from 2003 to 2016, which means that various localization algorithms will be presented in the next section. The advantage can help researchers quickly learn what types of methods have been used in UWSNs and which extent of the performance of various algorithms have been achieved. In addition, we classify the positioning algorithms based on different criterions. Here, we take five aspects into account, namely, computation algorithm, spatial coverage, range measurement, the state of the nodes and communication between nodes. In addition, much-related knowledge is contained in our paper such as acoustic communication, network architecture, routing techniques, challenges or open issues. The purpose of presenting our paper is that we hope a whole architecture of UWSNs will be built and provide a quick way to find the knowledge that the reader may need. In our paper, we concentrate on the localization algorithms and introduce numerous papers to show and compare with them. In addition, the comparison of survey papers for the localization algorithms in UWSNs is shown in Table 1.

Our paper concentrates on a comprehensive survey of positioning technology for UWSNs and is organized as follows: we introduce the acoustic communication, the network architecture and routing techniques in Section 2. Sequentially, the localization algorithm taxonomy is presented in Section 3. For each taxonomy, we describe the difference of the localization algorithms and then use the references to show positioning performance of this technology. We discuss in Section 4 and provide a qualitative comparison of the positioning algorithms. In Section 5, numerous challenges and open issues are described. The conclusions are presented in Section 6.

## 2. Related Knowledge

### 2.1. Acoustic Communication

Due to the underwater characteristics, both RF and optical signal are not suitable for UWSNs. Fortunately, the acoustic signal is suitable for estimating the position information in a marine environment. Usually, in UWSNs, the absorption of acoustic signal frequency is less than three orders of magnitude, which is acceptable [14]. Acoustic communication with omnidirectional transmission and a distribution channel seem good to use in UWSNs providing the low cost, ad hoc nature, and densely deployed application. However, acoustic communication has many challenges. In [15], the authors conclude these characteristics of the challenges:

- High propagation delay: The RF wave speed in the air is faster than that of acoustic waves underwater, which leads to the severe propagation delays [16].
- High energy consumption: The transmission power of UWSNs is superior to that of the terrestrial. In addition, the continuous movement of the underwater node may increase the energy.
- High noise and interference: The transportation and the movement of water currents can cause the noise during the acoustic communication. Meanwhile, various factors such as the animals, reflections resulting from the surface or bottom can bring interference.
- High dynamic topology: Because of the water current, the sensor nodes are not static, which means that the network topology is not fixed and changes with time.

### 2.2. Network Architecture

As we know, the energy consumption is important in UWSNs, which may limit the long lifecycle. Hence, the network topology is an essential aspect that needs to be well designed to decrease the severe impact on the network performance. In addition, both the network’s reliability and capacity rely on the topology. Therefore, how to organize such a network topology is a challenging job to which the researchers need to pay more attention. Here, we categorize the network architecture as 2D-UWSNs and 3D-UWSNs. For 2D-UWSN architecture: in [17], the authors conclude that this type of architecture is as follows: a group of sensor nodes (cluster) consists of UWSNs, and a cluster head (anchor node) is required for each cluster. Anchor nodes collect the information which is gathered by each member of the cluster and relays it to the surface buoyant nodes. The 2D communication contains a horizontal communication link and a vertical communication link. The communication between each cluster communicates, and the communication between related anchor node and the sensor node are horizontal communication links; however, the communication between the anchor node and surface buoyant node is a vertical communication link. 3D-UWSN architecture: some ocean phenomena cannot be adequately observed only by utilizing the sensor nodes in the ocean bottom. Hence, different depths have deployed the sensor nodes in the 3D-UWSN architecture. Each sensor node conveys the local underwater activity. In [2], three types of communications (3D) are described as follows: (1) inter-cluster node communication at different depths; (2) sensor-anchor node communication; and (3) anchor-buoyant node communication.

### 2.3. Routing Technique

Routing techniques are the guarantee for the reliable and efficient communication between the anchor nodes and the unknown sensor nodes [15]. The design of routing techniques in UWSNs may face more difficulty than the terrestrial environment. The mobility of the node with the water currents may lead to less reliability. In addition, the high propagation delay may decrease the effectiveness. Due to the difference, numerous valuable techniques of WSN cannot be directly employed into UWSNs. Therefore, many new techniques need to be redesigned and verified by using experiments. Various applications are limited because only the acoustic communication is suitable for UWSNs. Fortunately, efficient routing techniques can decrease the power consumption and improve the reliability of UWSNs. In this field, plenty of routing techniques have been reported [18,19,20,21,22,23]. Some survey papers have concluded routing techniques [7,14,15]. The authors in [7] classify protocols based on the energy-efficiency and reliability and consider protocols devoted to ameliorate energy-efficiency and reliability. The authors present various protocols and compare them in detail, while in [15], the authors describe the routing protocols in two aspects, namely, the cross-layer design routing and the non-cross-layer design routing. Different from the taxonomy of [15], the authors in [14] discuss these routing techniques based on localization, routing, and reliability. Three papers have collected numerous papers and made a detailed comparison. Therefore, we will not introduce the context of those routing techniques that can be found in the three papers.

## 3. Localization Algorithm

**Figure 1:**
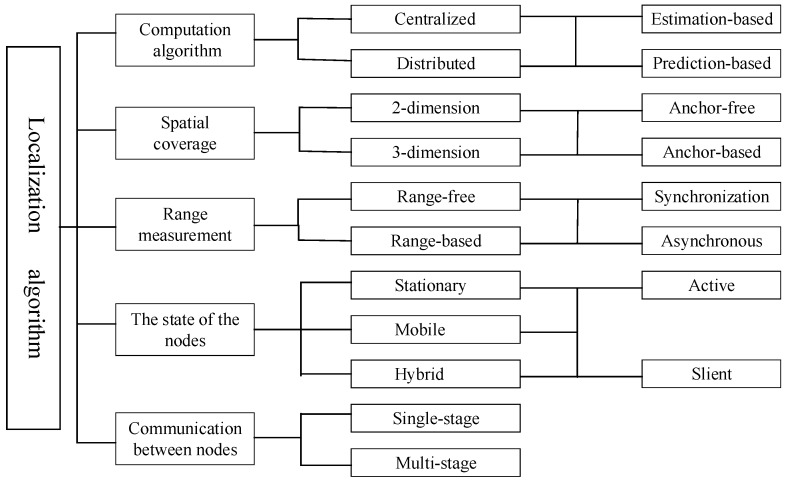
Taxonomy of the localization algorithms in UWSNs.

Localization has drawn a lot of attention from the researchers in UWSN research fields because the localization algorithms of WSN are not suitable for use in UWSNs directly. Hence, new methods or improved techniques must be put forward, and many research results have been reported. To make a better comparison of these papers, we classify them into five aspects, namely, computation algorithm, spatial coverage, range measurement, the state of the nodes and communication between nodes. We show the taxonomy figure in Figure 1. In the next section, we will introduce each part of the taxonomy in detail.

### 3.1. Computation Algorithm

Based on the computation algorithm to perform, we divide the computation algorithm into two classes, namely, centralized and distributed techniques. To put it simply, the centralized technique estimates the position of the sensor node at a sink node. Each sensor node does not need to know its own location. However, the distributed technique estimates the sensor node’s position individually. That is to say that each sensor node collects related information such as the distance to anchor, anchor position, the distance to neighbors or the angle information to realize localization, and then the localization algorithm is performed in each sensor node. Then, the centralized or distributed techniques are classified into two subcategories as estimation-based and prediction-based schemes. In the estimation-based method, the latest available information is used to obtain the current position of the sensor node. For the prediction-based scheme, the previous node position, the distance information, and the anchor positions are employed to predict the node position at the next time instant. Thus, it is suitable for use in a mobile UWSN or a hybrid UWSN.

#### 3.1.1. Centralized Technique

We have collected some articles based on the centralized technique that are classified into two subcategories, namely, estimation-based and prediction-based schemes.

As for the estimation-based scheme, researchers in [24] propose two different types of algorithms. In [24], a Reverse Localization Scheme (RLS) that has a fast reaction to events is proposed and suitable for 3D mobile UWSNs. In this scheme, the ordinary sensor nodes have mobility with the water currents so that the height of the nodes is not fixed and can be used to detect a phenomenon in a 3D-UWSN. The 3D localization is transferred into 2D localization by equipping the pressure sensor in the ordinary sensor nodes to obtain the depth information (z-coordination). Surface anchors nodes are employed to help to estimate the position of an ordinary node. The sink or base station has a higher process capacity. Therefore, the data can be sent there, and the localization algorithm is performed at the station.

Considering the prediction-based scheme, a collaborative localization algorithm that is self-organizing and autonomous is proposed in [25]. The nodes can collaborate to estimate their location information autonomously. The spatial variation of a process is sensed by the nodes and described as follows: starting at the surface, buoyancy is utilized to reach a deeper depth of the ocean and then float to the sea surface. Buoyancy repeats this process until a maximum desired depth is reached. The authors determine configurations of the swarm and use TOA measurements to obtain distance estimation. Those distance estimates are used to periodically position the profile concerning other nodes. Then, using a moving reference frame, the authors can estimate the absolution position of the sensor nodes.

#### 3.1.2. Distributed Technique

In this subsection, we discuss the distributed techniques in two aspects, namely, the estimation-based and prediction-based schemes. Similarly, we introduce the main algorithms of these types of techniques.

**Figure 2:**
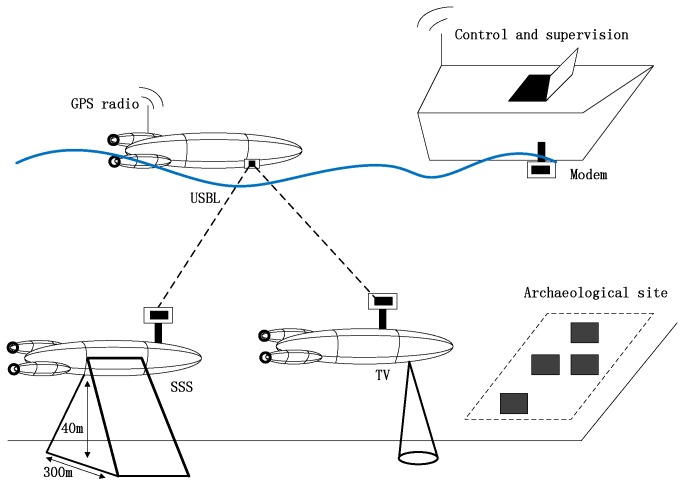
Typhoon AUVs of the swarm [6].

Firstly, we consider the estimation-based schemes. In [6], the authors present the experimental results obtained with two techniques for navigating an AUV within an acoustic sensors network by using a mixed USBL/LBL system. The vehicle employs the USBL measurements first to localize the fixed nodes and then to perform autonomous navigation in the first technique. The second technique can estimate both the navigation of the vehicle and the topology of the network by using the acoustic measurements. Furthermore, the authors called this framework Acoustic based SLAM (A-SLAM). The vehicle, called Typhoon, is able to cooperate in swarms to perform navigation, exploration, and surveillance of underwater archaeological sites. As shown in Figure 2, three different underwater vehicles (vision explorer, acoustic explorer, and team coordinator) cooperate as a single team, and each vehicle of the team can be customized for different mission profiles; moreover, the team composition can be altered. The algorithms have been tested on experimental data collected during the CommsNet13 campaign, led by the NATO CMRE in September 2013, with the Typhoon vehicle. The results show that the algorithm can achieve the error bounded within values suitable for most of the applications even with a low-cost, low-accuracy INS and acoustic positioning information coming at irregularly spaced.

**Figure 3:**
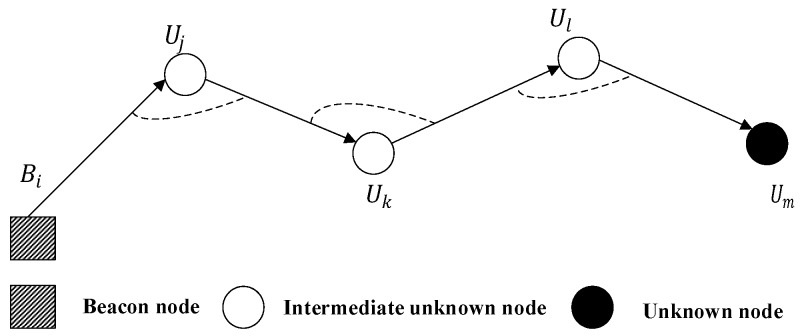
Polygonal line paths [26].

In [26], the authors take isolated unknown nodes into account. Considering the mobile UWSNs, the nodes are mobile with the water currents. Hence, the nodes may be dispersed and cannot communicate with enough beacons. The authors propose a Multi-hop Fitting Localization Approach (MFLA) that sets the intermediate nodes between beacons and unknown nodes as routers to construct paths (shown in Figure 3) via a greedy method, then fitting the multi-hop path into straight lines and estimating the position of the node through trilateration. When hidden nodes cannot find three neighboring beacons, MFLA will be executed. Then, some intermediate nodes are employed to form a polygonal line from the hidden nodes to the nearest beacons subsequently. This polygonal line is fitted for trilateration localization.

In [27], a Localization with Directional Beacons (LDB) for use in 3D UWSNs is proposed. The AUV acts as the beacon sender to estimate the locations of the sensor node. Hence, the AUV broadcasts beacons towards the sensor nodes at a constant interval. The nodes silently listen to the beacons. After two or more beacons are received, the nodes can estimate the positioning information. Those nodes hearing the beacon and the beam form different circles of different *h*. The circle center is $\left(x,y,h\right)$. Hence, the circle radius can be expressed as follows:

(1) r=tan(α/2)×Δh,

where α is the angle of the conical beacon and $\Delta h=|h_{A}-h|$. Through the receiving beacons, the rough position $\left(x,y,h\right)$ can be obtained. In the LDB scheme, each node uses received beacons from the AUV to obtain its positioning information independently.

In [28], a Localization with a Mobile Beacon (LoMoB) is proposed. The LoMoB is an improvement of LDB. The mobile beacon that knows its location broadcasts a beacon containing its positioning information with constant distance intervals. The sensor nodes within communication range can receive the beacon. Similarly, the LoMoB has a beacon point selection process.

**Figure 4:**
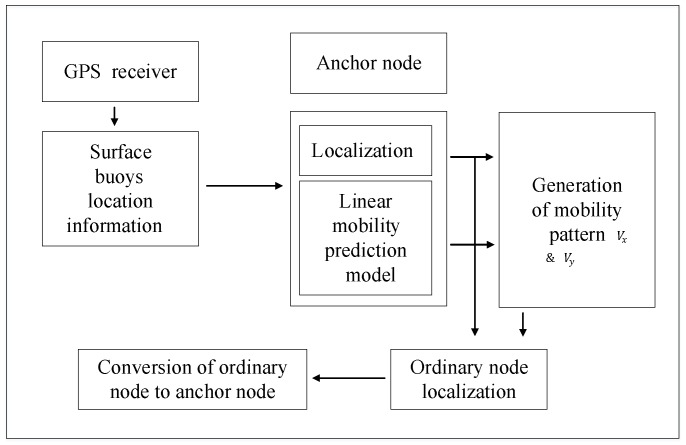
Block diagram of the proposed EMBL algorithm [29].

As for the prediction-based scheme, the authors in [29] propose an Efficient Mobility Based Localization (EMBL) scheme. In this scheme, the prior known positioning information of the node is used to predict its future mobility pattern and obtain its position at the next time. The kinematic mobility model is employed in this scheme to represent the movement in UWSNs. The authors describe the particular algorithm in Figure 4. Anchor nodes with more energy can position themselves in each localization period, and they perform the complex mobility prediction algorithm. The localization of ordinary nodes contains the mobility prediction and location estimation. A Scalable Localization scheme with Mobility Prediction (SLMP) is presented in [30]. The localization process is similar with the EMBL.

In this subsection, we have discussed the computation algorithm and the difference between the centralized and distributed techniques. We use some papers to show the different characteristics of the two techniques. Furthermore, we summarize these papers that use the computation algorithm to achieve the positioning in Table 2. We mainly compare the coverage, localization time, accuracy, computational complexity, and energy consumption. It is not suitable to discuss what the best algorithm is when the application scenario is not taken into account. For example, if the power is limited, the reader should choose the localization algorithm with low energy consumption. If high accuracy is required, the reader should choose the localization algorithm with high localization accuracy, but, at the same time, the computational complexity may be high. Thus, according to the following table, the reader can choose the suitable algorithm for doing some application or doing some improvement. The comparisons between the localization algorithm referred to in this subsection and other localization algorithms that are introduced in other subsections will be shown in Section 4.

### 3.2. Spatial Coverage

We consider the spatial coverage as two types, namely, 2D-networks and 3D-networks, which are the same as the network architecture of UWSNs. This taxonomy mainly focuses on which architecture of the localization algorithm is used or to what extent spatial coverage can be achieved. 2D-networks or 3D-networks are classified into two subcategories as anchor-free and anchor-based schemes. In UWSNs, a lot of localization schemes may employ the anchor node to help realize localization. However, some researchers also propose self-localization algorithms that do not need the anchor node.

#### 3.2.1. 2D-Networks

As we presented before, 2D-networks are classified into two subcategories, namely, anchor-free and anchor-based schemes. However, unfortunately, we cannot find the paper that belongs to the anchor-free 2D-network subcategories, so we just introduce some papers belonging to the anchor-based 2D-networks. In [31], the authors propose an Underwater Tracking (UT) scheme that can also be called as Drift-dependent UT (DD-UT). In addition, the effects of the ocean current and sound-speed uncertainties are considered. Acoustic communication reports the drift velocities of the anchors that are used to obtain the drift velocity of the Tracked Node (TN). Using the Unscented Kalman Filter (UKF) and Extended Kalman Filter (EKF), Two State-Space Model (SSM)-based tracking solutions can be obtained. It is proved that UKF is more robust and superior for the complex cases than EKF when a large amount of data is available.

The authors in [32] propose two types of localization schemes in this paper, which are fixed-position and magnified-range. The fixed-position can ameliorate connectivity of the system. In this paper, the fixed-location nodes mean that the node will not move with the target, but not to say that they are anchored in a particular position. They can move in a predefined area. Thus, the design of the fixed-position scheme can reduce the power consumption. The magnified-range is based on fewer energy constraints of anchor nodes. All regular sensor nodes can connect to anchor nodes directly or indirectly. In addition, the magnified anchors’ signal range will improve the likelihood of obtaining more accurate results.

#### 3.2.2. 3D-Networks

**Figure 5:**
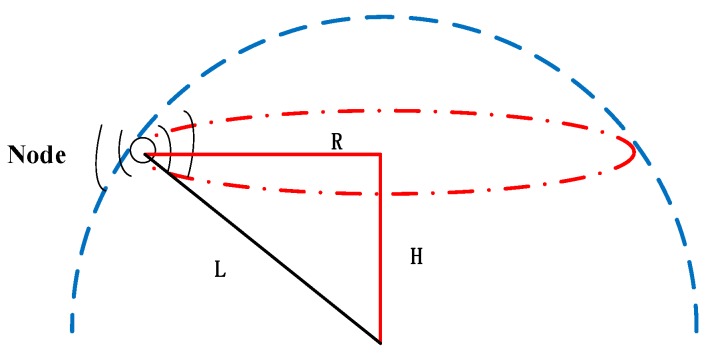
Model of an active-restricted underwater sensor node [33].

Similarly, 3D-networks are classified into two subcategories, namely, anchor-free and anchor-based schemes. Firstly, we introduce the anchor-free scheme. An Anchor-Free Localization Algorithm (AFLA) is presented in [33], which is suitable for the active-restricted UWSNs. This algorithm takes advantage of the relationship of adjacent nodes and does not require an anchor node. In this scheme, the active-restricted nodes mean, when they anchor at the bottom of the sea, they can float in the sea and move within a hemisphere area (shown in Figure 5). The anchor and the cable length (denoted as L) is the center and radius of the hemisphere, respectively. Note that the pressure sensor can obtain the depth (H) of the node. The node with the unknown location broadcasts its spherical center’s coordinates, depth, and cable length. When two messages are received by the node, it calculates the position. In addition, the authors consider the various characteristics of the mobile network and describe the mobile node localization process in detail.

**Figure 6:**
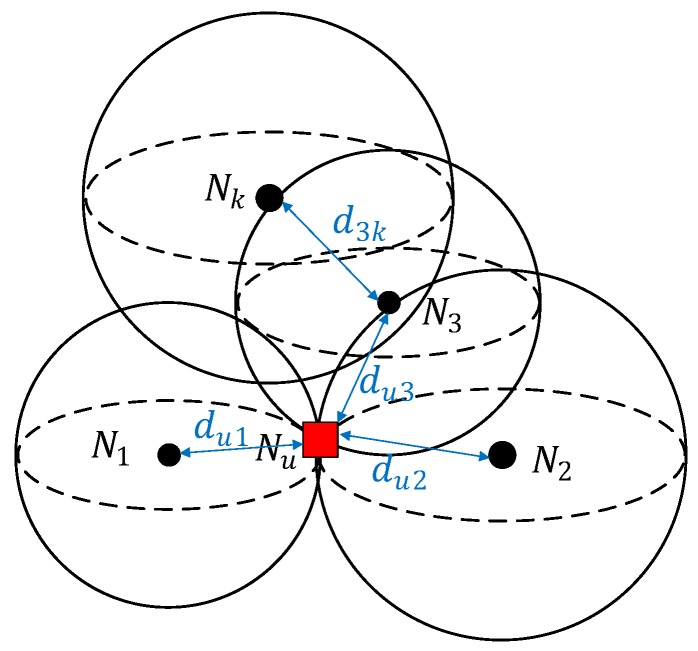
Node multihop localization [34].

As for the anchor-based scheme in 3D-UWSNs, a relatively larger amount papers will be introduced. In [34], the authors analyze the problem existing in the nonlinear least-square-based node self-localization scheme, point out the biased distribution of multi-hop distance estimation error, and employ the orthogonal regression method to solve the problem of normal nodes localization in the case of error anchor position. The multi-hop localization framework is shown in Figure 6. The normal node and anchor node can be denoted as $N_{u}$ and $N_{a}$, and their coordinates are $X_{a}={\left[x_{u},y_{u},z_{u}\right]}^{T}\in R^{3}$ and $X_{a}={\left[x_{a},y_{a},z_{a}\right]}^{T}\in R^{3}$,respectively, where a=(1,2,…,K), and *K* is the anchor node number. $N_{u}$ can obtain all multihop distance estimations to the anchor nodes by multihop information exchange. The problem of locating the node $N_{u}$ can be shown as the following nonlinear regression model:

(2) $$d_{a}+e_{a}=\left|\right|X_{u}-X_{a}|{|}_{2},$$

where $X_{u}$ and $X_{a}$ are the regression parameters and the independent variables, respectively. Observations of $X_{a}$ are anchors’ declared coordinates $\left\{X_{1}^{\prime },X_{2}^{\prime },\ldots ,X_{K}^{\prime }\right\}$. $d_{a}$ is the dependent variable, and its observations are the estimated distances between $N_{u}$ and $N_{a}$. $e_{a}$ is the distance estimation error. Then, the Nonlinear Least Squares Estimator (NLSE) can be used to solve the unknown parameter $X_{u}$:

(3) $$\begin{array}{cc} {\hat{X}}_{u} & =\arg\min_{X_{u}}\sum_{a=1}^{K}{\left(e_{a}\right)}^{2}\\ & =\arg\min_{X_{u}}\sum_{a=1}^{K}{\left(||X_{u}-X_{a}|{|}_{2}-{d^{\prime }}_{a}\right)}^{2}, \end{array}$$

where ${\hat{X}}_{u}$ are the estimated coordinates of $N_{u}$.

Two sources of distance estimation errors of the multi-hop scenarios are discussed and empirical analysis of multi-hop distance estimation bias for 3D-UWSNs is given in this paper. To address these issues, an anchor position error-tolerant multi-hop localization method based on the orthogonal regression for UWSNs is proposed.

In [35], a localization scheme is proposed that only requires two references to approximate locations that are suitable for use in low-density deployment. It is a Range-based Low-overhead Localization Technique (RLLT). The anchor node broadcasts its position, and the nodes within the transmission range of the anchor estimate the distance from the anchor. However, there still some nodes that are out of the transmission range of an anchor. Hence, in this scheme, once a node is located, it becomes a new reference node and broadcasts its position. When a node can obtain some distances from anchor nodes, it uses those distances to calculate its position.

**Figure 7:**
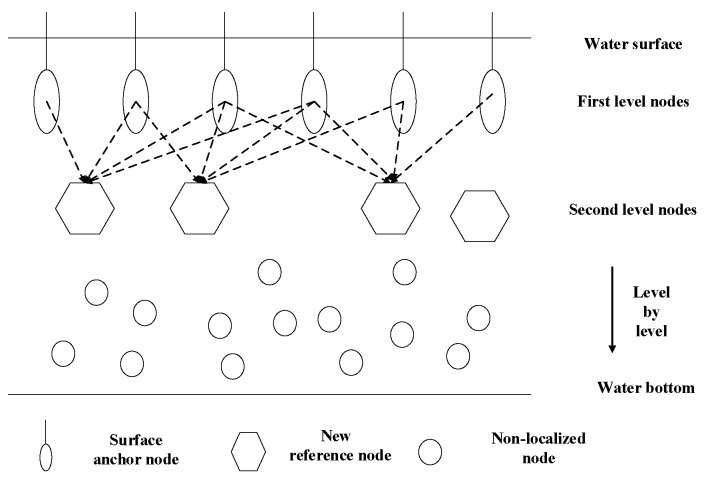
Network architecture of TPS [36].

In [36], a Top-down Positioning Scheme (TPS) that is suitable for UWSNs increases the localization coverage while keeping the localization error small. The node deployment of this scheme is shown in Figure 7. Firstly, only the ordinary nodes close to the surface anchors can obtain the location information, and they calculate the confidence values and compare with the threshold of confidence. They become the new reference nodes only when the confidence values of the nodes are larger than the confidence threshold. The localization algorithm is the gradient method and a scheme that can estimate two-hop Euclidean distance in 3D space is proposed to help the non-localized node find enough reference node to realize localization.

Based on the color filtering technology, the Projection-color Filtering Localization (PCFL) algorithm and the Anchor-color Filtering Localization (ACFL) algorithm are shown in [37]. Both algorithms can provide precise localization with minimum power wastage. The detailed localization process can be described as follows: firstly, reconstituting the present network construction as a hierarchical structure, the authors describe the localization as a geometry problem. Secondly, task projections are considered as a center. Hence, the task-rings can be obtained. PCFL is based on the Angle of Arrival (AOA) measurement and the task projections obtain the original RGB values, while, for ACFL, and the task anchors obtain the initial RGB. Finally, the nearness degree is defined as a filter samples, and, meanwhile, it is stored as a weight.

In this subsection, we have discussed the spatial coverage and the difference between 2D-networks and 3D-networks. We use some papers to show the different characteristics of the two network architectures of UWSNs. Furthermore, we summarize these papers that use the spatial coverage to achieve the positioning in Table 3. We mainly compare the coverage, localization time, accuracy, computational complexity, and energy consumption. According to the following table, the reader can choose the suitable algorithm for doing some application or doing some improvement. The comparisons between the localization algorithm referred to in this subsection and other localization algorithms that are introduced in other subsections will be shown in Section 4.

### 3.3. Range Measurement

According to the range measurement, we divide the localization algorithms into two classes, namely, range-based scheme and range-free scheme. Generally speaking, the ranged-based scheme estimates the distance and then converts it into the position information. The algorithm contains the TOA, Time Difference of Arrival (TDOA), AOA and Received Signal Strength Indicator (RSSI). In [38], the authors describe the range-based scheme as three steps, namely, range measure, location estimation, and calibration. Two cases of range measurements are discussed, namely, reference node within transmission range of the ordinary and reference node outside the transmission range of the ordinary. It is suggested that RSSI, TDOA, and TOA are suitable for ordinary nodes to estimate their distances to the reference nodes, while, for the second case, the Euclidean distance propagation method is more suitable. In the phase of location estimation, at least d + 1 range measures are needed to estimate the location of the d-dimensional space. The aim of calibration is to refine the location estimation. The range-free scheme does not need the range measurement and bearing information, and employs the local topology and locations of the surrounding anchor nodes to estimate the location. This scheme can only obtain a coarser position, that is to say that the precision is lower than the range-based scheme. Then, we continue to classify range-free or a range-based algorithm into two subcategories as synchronization and asynchronization schemes. As we know, the time synchronization is an important factor to pay attention. In addition, in many cases, the assumption is made that sensor nodes are synchronized with each other. Because of the difficulty of the precise time synchronization, some localization algorithms are proposed without the synchronization requirement.

#### 3.3.1. Range-Free Scheme

**Figure 8:**
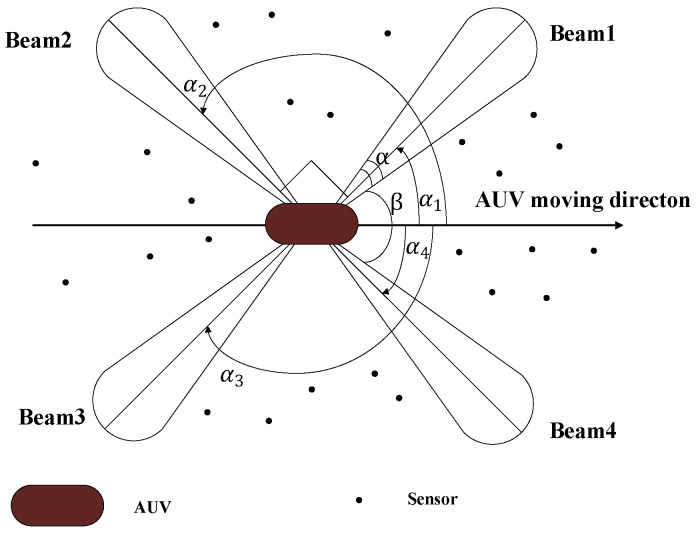
System architecture contains sensor nodes and a mobile AUV equipped with four directional beams [39,40].

In this part, we discuss the range-free scheme where the range between the nodes is not required without the synchronization requirement. On the contrary, the range measurement method such as TOA, TDOA needs the synchronization requirement. Thus, we introduce some papers that are asynchronous based on a range-free scheme. In [39,40], the range-free scheme employs an AUV to periodically broadcast a message block via four directional acoustic beams. Both AUV position and a directional dependent marker are contained in the message block. The directional dependent marker is used to identify the respective transmit beam. As showed in Figure 8, the angles between the beams and the AUV body are fixed. The node receives the message and using the two different successive beams can obtain the location of the AUV at two different time instants. Then, utilizing the two estimated positions can obtain the position of the node.

Considering that many applications do not require accurate position information, the authors in [41] present an efficient Area Localization Scheme (ALS). This scheme localizes the sensor within a certain area. Hence, this scheme is simple because no range measurement needs to be made in the sensor node, and the complex calculation deals with powerful sinks. The anchor nodes broadcast beacon signals and the acoustic signals can be sent at varying power levels. The sensor nodes passively listen to the signals and record the information. According to the fact that the signal levels from the same anchor node are different at different locations of the sensor node. The sensor node measures its signal coordinates and stores this information. The sensor node forwards the stored information to the sinks when required. Then, the sink utilizes the information collected from the sensor nodes that can localize the area where the sensor node is located.

#### 3.3.2. Range-Based Scheme

The range-based scheme is discussed in two aspects, namely, synchronization and asynchronization. Firstly, we present some papers to introduce the synchronization scheme. In [42], the localization in UWSNs is classified into four steps, namely, node discovery, range measurement, data filter, and location calculation. In the node discovery step, the active sensor node measures the range from the neighboring nodes. In the second phase, the nodes send data to the gateway buoy after all nodes have finished the distance measurement. Then, all data are sorted into a stack by the gateway buoy. In the third step, the statistical theories are employed to help the gateway buoy filter all measured data. After that, the results are a 2D matrix. Finally, the algorithm first calculates the position of the unknown node. After all unknown nodes are finished, the iterative process starts. The algorithm has some similar point with the greedy algorithm.

**Figure 9:**
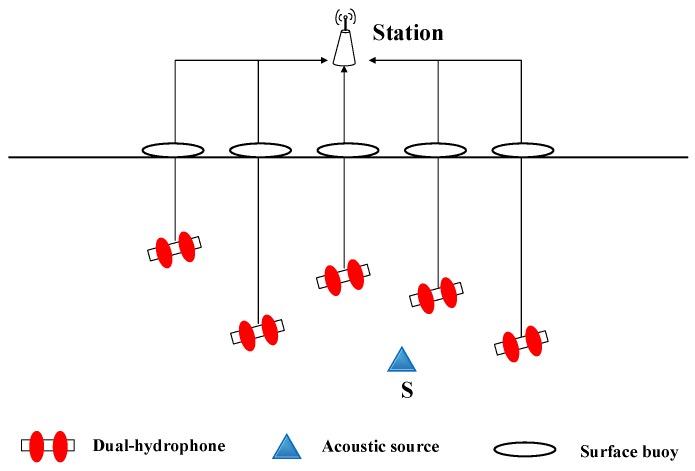
System overview. A 3D-UASN formed by randomly deployed dual-hydrophone nodes, which can be used to locate the acoustic source. Each node floats with a buoy [43].

In [43], a Dual Hydrophone Localization (DHL) method converts the localization problem into half-plane intersection issues. In this scheme, the network consists of an acoustic source (S), some surface buoys and some dual-hydrophone nodes (shown in Figure 9). The dual-hydrophone nodes connect with the surface buoy by a cable. The buoy sends the sensed data that is received by the nodes to the central station, and then the station calculates the coordinates of the acoustic source by analyzing the data. The beep emitted by the acoustic source propagates along the cable. The node within the sensing range will receive the signal, and then the Generalized Cross Correlation (GCC) algorithm is employed to compute the TDOA from S to its dual-hydrophone. For the ith node, we draw a perpendicular plane to classify the whole localization area into two regions. Then, the node judges the source location to the right or left of the perpendicular plane and marks with binary code 0 or 1. A station gathers and analyzes the binary information from the sensor nodes and draws the feasible (polyhedron) region of S. We take the polyhedron center as the estimated location of the acoustic source.

In [44], a Sequential Time Synchronization and Localization (STSL) algorithm in UWSNs is proposed. The packet exchanges between the anchor and non-localized nodes are employed in this scheme. Hence, to get accurate short-term motion estimation, the scheme takes advantage of the directional navigation systems used in nodes. The STSL algorithm consists of two steps, namely, time-synchronization step and the localization step. The first step is to provide estimations of the propagation delays that are accomplished by two-way packet exchange. In the localization, the location of the non-localized nodes can be obtained.

Sequentially, we introduce some papers that are asynchronous based on a range-free scheme. The issue of asynchronous clocks being located in UWSNs is considered and an On-demand Asynchronous Localization (ODAL) scheme is proposed in [45]. The authors use the sequential transmission protocol and the broadcast nature of the acoustic signal to locate the network with a small overhead. The localization protocol is executed in the initiator node by broadcasting to initiate a transmission sequence with the anchor node. In the localization broadcast procedure, anchor nodes collect and forward information. Passive nodes silently listen to the broadcast messages and localize themselves.

In [46], an Underwater signal Reflection-enabled Acoustic-based Localization (UREAL) scheme is proposed. It makes use of the multimodal directional underwater piezoelectric transducers that are used to generate either omnidirectional or directional beams. Therefore, the proposed scheme can utilize both Line-of-sight (LOS) and surface-reflected Non-line-of-sight (NLOS) links to realize the localization of nodes. UREAL uses RSS information for LOS/NLOS link taxonomy, while the AOA ranging is used in the position estimation.

A GPS-free passive localization scheme is presented in [47]. The range data are employed to estimate the position information of the sensor nodes based on the planar trigonometry principles. Localization process with the Seaweb server contains five steps, and, once the node is localized, we classify the whole network field into circular levels and sectors. The aim is to lengthen the lifetime of the sensor node and decrease the traffic complexity. The mesh network is formed inside each of the sectors, which increases the reliability.

**Figure 10:**
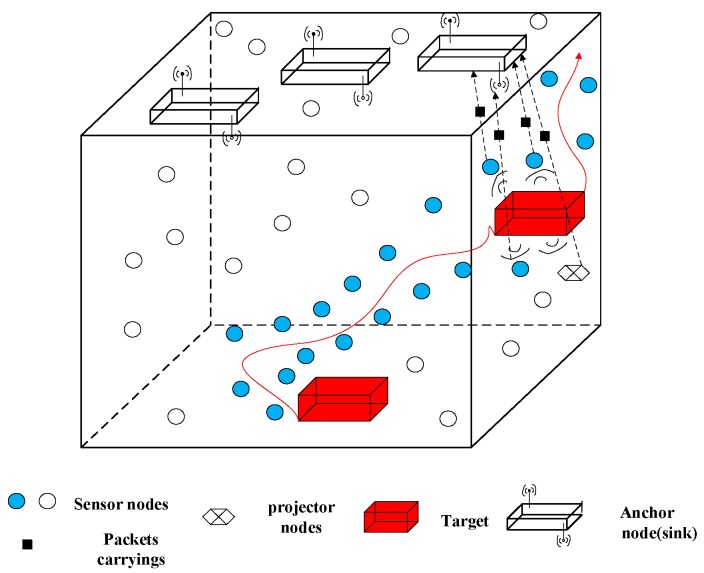
A network model for UWSNs that implements 3DUT [48].

In [48], the authors propose a 3D underwater target tracking (3DUT) scheme to collaboratively complete the underwater target accurate tracking with the least energy consumption synergy. The scheme is based on the TOA algorithm and using the trilateration to obtain the positioning information of the target. Making the most of the positioning information and the calculated velocity of the target, and employing a new target-movement-based duty-cycle mechanism, can achieve the energy-effective target tracking. Due to the continuous surveillance, the energy resources of boundary nodes may deplete rapidly. Considering the problem, the authors employ an adaptive procedure to find, designate, and activate new boundary nodes. The network model is shown in Figure 10. In this scheme, at least three anchor nodes are required. Hence, the sink gathers the information and performs the calculations. The sensor nodes collect and send data to the sink. The projector node may change during the tracking process.

In this subsection, we have discussed the range measurement and the difference between range-based schemes and range-free schemes. We use some papers to show the different characteristics of the two schemes. Furthermore, we summarize these papers that use the range measure to achieve the positioning in Table 4. We mainly compare the coverage, localization time, accuracy, computational complexity, and energy consumption. According to the following table, the reader can choose the suitable algorithm for doing some application or for doing some improvement. The comparisons between the localization algorithms referred to in this subsection and other localization algorithms that are introduced in other subsections will be shown in Section 4.

### 3.4. The State of the Nodes

According to the state of nodes, we classify UWSNs into three taxonomies, namely, stationary, mobile and hybrid UWSNs. The feature of the stationary network is the node fixed at a particular location. Hence, it can be used to monitor a given region. In the mobile network, the mobility of the node can be free or controlled. In [11], propelled equipment such as AUVs and Unmanned Underwater Vehicles (UUVs) can control the mobility of the nodes. As for the hybrid networks, the stationary and mobile nodes coexist. Then, we continue to classify stationary, mobile, and hybrid networks into two subcategories as active and silent messaging properties. The silent messaging property means that only the anchors can send the localization messages, but the underwater nodes can just receive but not send, while the dynamic messaging property is both the anchors and underwater nodes that can send a message to realize localization.

#### 3.4.1. Stationary

We first consider the active messaging properties based on the stationary state and collect several papers to describe it. In [49], an Asymmetrical Round Trip based Localization (ARTL) algorithm is presented. The algorithm with excellent scalability does not require time synchronization. In this scheme, there exists only one pair of message exchanges and the others passively listen to the beacons. The authors in [50] consider the direction of the sensor node and the beam width and develop a Loop Assisted Synchronization-free localization Algorithm (LASA) to achieve a synchronization-free localization algorithm. This algorithm obtains the distance measure through the two-way range TOA. The link status detection and the symmetric links ranging is completed with the help of a link detection stage. Then, the first part of ordinary nodes is located through the anchor nodes, while the other ordinary nodes obtain the positioning information via an iterative method. Finally, the loop-assist method is employed to locate those nodes whose positioning information is still unknown.

A reactive localization scheme proposed in [51] can be described as three phases, namely, finding the anchor nodes, reactive localization of sensor nodes, and delivery of information. The first step is finding a subset of anchor nodes to ensure that every sensor node is covered by four non-coplanar anchor nodes. In the second phase, a sensor node detects an event and broadcasts a message. After the anchor nodes receive the messages, they send their positioning information as a response. In the third step, the node transmits messages that contain its position and the sensed information to the sink.

As for the silent messaging properties based on the stationary state, we still introduce some papers to show it. In [52], an Underwater Sensor Positioning (USP) scheme is proposed. Once two distance information from two anchor/reference nodes is obtained, the to-be-localized sensor node computes its position. If two distances are not obtained, its position can be iteratively resolved.

A cubescan-based 3D multi-hop localization algorithm presented in [53] is suitable for large-scale UWSNs. The scheme is described as three steps. Firstly, the authors restrain the location of the unknown sensor node in a feasible set. Secondly, the authors construct a weighted constrained multi-hop localization model by studying the factor that may influence distance estimation of the multi-hop localization. Thirdly, dividing the corresponding geometrical intersection into a number of equal size cubes search a certain center as the final results.

In [54], a time synchronization-free localization algorithm is proposed. This algorithm contains two steps, namely, distance measurement and position estimation. In the first step, the two messages received from the mobile beacon at a different time are used to measure the distance from the sensor node to that mobile beacon. Then, three distances of the sensor node from three different mobile beacons can be obtained. In the second phase, forming a nonlinear equation system by using the three distances can obtain the coordinates of the sensor nodes.

#### 3.4.2. Mobile

We still first consider the active messaging properties based on the stationary state and collect several papers to describe it. In [55], the time synchronization and localization based on the semi-periodic property of seawater movement scheme is called SLSMP. The deployment is described as follows: the reference nodes attached to the buoys and the sensor nodes equipped with tiny gyro and acceleration sensors are used. The reference nodes can obtain the global time and real-time position on the GPS. The SLSMP protocol contains three phases, namely, Sending Point (SP) selection based on trajectory tracking, message exchange, and time synchronization/localization. In addition, the knowledge about sensor deployment, inertial navigation system (INS) and filter technique is employed to ameliorate the localization accuracy.

A Joint Solution for Localization (JSL) is shown in [56]. This scheme consists of four steps, namely, data collection, synchronization, localization, and iteration. In the first step, data collection means that an ordinary node can obtain the reference time and positioning information. In the second period, based on the information obtained in the first phase, the ordinary node performs the synchronization process. In the third step, based on the estimated propagation delays, the localization process is carried out. In the fourth phase, the iteration process acts as if the estimated position in the third stage is regarded as the input to the second phase to update the rough position in the next round of message exchange.

In this part, we discuss the silent messaging properties based on the mobile state. A multi-stage localization scheme that employs mobile beacons into UWSNs is presented in [57]. Mobile beacons periodically rise and dive in the underwater. GPS coordinates will be received when mobile beacons resurface. Then, these mobile beacons fall underwater to broadcast these coordinates. If the sensors are localized, the sensors are looked at as the proxy beacons and broadcast their positions. In this method, the authors use proxies instead of the beacons to minimize the number of the beacons.

In [58], the authors employ an AUV aiding in the localizing process and the priority infrastructure or synchronization between nodes are not required. In this scheme, three messages, namely, wake-up, request and response messages are contained. Because of asynchronization between the nodes, the authors use the request/response message pair to measure the Round Trip Propagation Delay (RTPD). First, the AUV sends a wake-up beacon to the sensor node and the sensor node that is in the communication range receives the wake-up message and then starts to measure range by sending a location request packet. When the AUV received the request, it replies a response packet with its coordinates. The authors consider two methods, namely, triangulation and bounding box. In the triangulation method, obtaining n-dimension coordinate needs n+1 equations and then solving the linear independent equations can get the estimation of the sensor location. Because the z-coordinate of the sensor can be obtained from the pressure sensor, we need three messages to estimate the 2D positions of the nodes and can be described as

(4) $${\left(x-x_{i}\right)}^{2}+{\left(y-y_{i}\right)}^{2}+{\left(z+z_{i}\right)}^{2}=d_{i}^{2},$$

where x,y,z are the coordinates of the unknown sensor node. $x_{i},y_{i},z_{i}\;\;\left(i=1,2,3\right)$ are the AUV coordinates. $d_{i}^{2}$ is the square of the measured range. The boundary method draws a rectangular area with the intersection of the estimated distances. The intersection of the diagonal lines indicates the position of the sensor node.

#### 3.4.3. Hybrid

In this part, we still first consider the active messaging properties based on the hybrid state. In [59], a 3D Underwater Localization (3DUL) algorithm is shown. Three anchor nodes float at the surface. It is a distributed and iterative algorithm and does not require time synchronization. Three surfaces’ buoys are employed for localization initially. The 3DUL can be described in two steps. In the first step, the sensor node whose location is unknown determines the distances to neighboring anchors. In the second step, the anchor is projected into the horizontal level for which the unknown sensor node is located by utilizing the pairwise distances and depth information. After the location of the sensor node is known, it becomes an anchor and helps to locate other nodes.

Then, we discuss the silent messaging properties based on the hybrid state. In [60], the authors present a silent positioning method. Both a pressure sensor and an accelerometer are equipped with an each sensor. Hence, the purpose of using a pressure sensor is the same as with other algorithms to measure the depth estimation. The accelerometer is used for sensor orientation estimation. In addition, the EKF is employed to overcome the state estimation problem. In [61], a range-free localization scheme is proposed. This scheme first finds two estimated locations of the sensor node by geometry. Finally, one estimated location is selected as the final results. The sensor node locates itself without any help from the other nodes and consists of two steps, namely, selecting three beacon points and estimating the location of a unknown node. In the first steps, the sensor node within the transmission range can receive the beacons from the mobile beacon, and the received beacons are projected in the plane parallel to where the sensor node is located. The first received beacon is selected as a beacon point, and the last received beacon within a predefined time is selected at another point beacon. Hence, this scheme needs at least three beacon points. In the second phase, three beacon points are not exactly on the projected transmission circle of the sensor node. Considered that the beacon distance is *d*, and the projected transmission range of the sensor node is $r^{\prime }$, then distance points are located between the distance $r^{\prime }-d$ and $r^{\prime }$ from the sensor node. The authors use the middle value of the range, i.e., $r^{\prime }-d/2$, to minimize the error of the estimated distance. Hence, solving the following equations can obtain the estimation location of the sensor node:

(5) $$\left\{\begin{array}{c} {\left\{\left(x-x_{1}\right)\right.}^{2}+{\left(y-y_{1}\right)}^{2}={\left(r^{\prime }-d/2\right)}^{2},\\ {\left\{\left(x-x_{2}\right)\right.}^{2}+{\left(y-y_{2}\right)}^{2}={\left(r^{\prime }-d/2\right)}^{2},\\ {\left\{\left(x-x_{3}\right)\right.}^{2}+{\left(y-y_{3}\right)}^{2}={\left(r^{\prime }-d/2\right)}^{2}, \end{array}\right.$$

where (x,y) are the coordinates of the sensor node, and $\left(x_{i},y_{i}\right)$ are the coordinates of the beacon $B_{i}$. Two intersection points of the two equations can be obtained and described as $P_{m}$ and $Q_{m}$. Hence, the third equation is needed to determine which ones are the true coordinates of the sensor node. If $\;||B_{3}-P_{m}\left|\right|-\left(r^{\prime }-d/2\right)\;\;|<|\;\;||B_{3}-Q_{m}\left|\right|-\left(r^{\prime }-d/2\right)|$ is satisfied, the location $P_{m}$ is used for the estimation of the sensor node; otherwise, select the $Q_{m}$.

In this subsection, we have discussed the state of nodes and the difference between the stationary UWSNs, mobile UWSNs, and hybrid UWSNs. We use some papers to show the different characteristics of the three kinds of UWSNs. Furthermore, we summarize these papers that use the state of nodes to achieve the positioning in Table 5. We mainly compare the coverage, localization time, accuracy, computational complexity, and energy consumption. According to the following table, the reader can choose the suitable algorithm for doing some application or doing some improvement. The comparisons between the localization algorithm referred to in this subsection and other localization algorithms that are introduced in other subsections will be shown in Section 4.

### 3.5. Communication between Nodes

According to the feature of communication between reference nodes and ordinary nodes, we classify the localization algorithms of UWSNs into two taxonomies, namely, single-stage scheme and multi-stage scheme. The single-stage scheme is that all the ordinary nodes exchange the message directly with the reference nodes. When they are localized, they remain passive and cannot help obtain the location information of the unknown nodes [38]. As for the multi-stage scheme, the ordinary node may not communicate with the reference node directly. Once the ordinary node is localized, they can be looked at as the new reference nodes and help to realize localization of the other ordinary nodes [38].

#### 3.5.1. Single-Stage Scheme

We describe the single-stage communication between nodes in this part. In [62], the proposed scheme utilizes the hyperbola-based approach for event localization and a normal distribution for estimation error modeling and calibration. Between the common circle-based and hyperbola-based method, there is some difference. Firstly, the two hyperbolas always have one intersection, but the circle-based method does not. In addition, for the hyperbola-based method, it is easy to find partial solutions that are used in the calibration process to further ameliorate the localization precision. However, for the circle-based method, adopting a partial differential approach or the matrix approach is needed.

In [63], the authors propose a Wide coverage Positioning System (WPS). Firstly, the authors consider relying on four anchor nodes, and the UPS scheme cannot uniquely localize the sensor nodes in the enclosed area and the sensor nodes residing close to the anchor nodes require five anchors. In WPS, the authors define that the using four anchors as UPS (4) can attain unique localization—similarly, if five anchors as UPS (5) are used. UPS (4) and UPS (5) are used together in which four anchors are already localization. In the WPS, if a message cannot be received, the sensor node should wait for a timeout period and then start re-initiating the positioning process. Compared with the UPS, the WPS performs better in providing higher unique localization success but with higher localization delay and communication cost.

#### 3.5.2. Multi-Stage Scheme

Similarly, we introduce several papers to start the multi-stage communication between nodes. In [64], the authors propose a hierarchical approach. First, all anchor nodes label themselves as references and confidence values are set to 1. The non-localized nodes are regarded as ordinary nodes. All nodes periodically broadcast the beacon message that contains its id. Hence, all neighboring nodes can receive the beacon message that can be used to measure their distances to this node. In this localization process, the ordinary node is localized and becomes the reference node. Then, the authors extend the Euclidean distance propagation method to adopt the 3D environment.

A framework that can establish localization and routing is presented in [65], and is suitable for mobile UWSNs. In this scheme, the localization and routing are done in two consecutive rounds, which means that the localization messages are used in the routing decision. In addition, the authors take the problem of decreasing the number of Mobile Beacon and Sink (MBS) nodes into account and then employ the iterative localization—that is to say that the localized node is active and broadcasts its coordinates. The “catch up or pass” forwarding algorithm that belongs to the greedy geo-forwarding scheme is proposed.

In this subsection, we have discussed the communication between nodes and the difference between single-stage schemes and multi-stage schemes. We use some papers to show the different characteristics of the two schemes. Furthermore, we summarize these papers that use communication between nodes to achieve the positioning in Table 6. We mainly compare the coverage, localization time, accuracy, computational complexity, and energy consumption. According to the following table, the reader can choose the suitable algorithm for doing some application or doing some improvement. The comparisons between the localization algorithm referred to in this subsection and other localization algorithms that are introduced in other subsections will be shown in Section 4.

## 4. Discussion

We have collected numerous papers that are positioning algorithms in UWSNs and introduced them to different aspects. The aim is to emphasize the different unique properties of the different aspects, which are computation algorithm, spatial coverage, range measurement, the state of the nodes and communication between nodes. Some papers belong to the corresponding subcategory. Actually, almost all of those papers have all aspects, and then we conclude which subcategories they really belong to in Table 7. However, if some subcategories are not shown in the references, we use “Not” to indicate it. In addition, we use the abbreviations to express all aspects, namely, Reference (Ref.), Centralized or Distributed (C or D), Estimation-based or Prediction-based(E or P), 2-Dimensional or 3-Dimensional (2D or 3D), Anchor-free or Anchor-based (Af or Ab), Range-free or Range-based (Rf or Rb), Synchronization or Asynchronization (Sy or As), Stationary or Mobile or Hybrid (Sa or M or H), Active or Silent (A or Si), and Single-stage or Multi-stage (Ss and Ms). Then, the symbol (√) listed below each subcategory means the corresponding subcategory.

## 5. General Challenges and Open Issues

The localization in UWSNs suffers more challenges than the terrestrial environment. The mobile current, the effect of transportation and other factors lead to the localization problems in the positioning algorithms. Here, we address some main challenges and open issues.

**Sound speed variation**: In many localization algorithms, the authors made an assumption that the sound speed is a constant. Not only the pressure and temperature but also the salinity of the sea water may affect the sound speed. In the multilateration algorithm, because of the assumption of the sound speed, the estimation of the coordinates of the sensor node may incorporate an error. Thus, how to construct a common and accurate sound speed model is a question.

**Unpredictable underwater environment and intricate network design and deployment**: Many aspects will affect UWSNs. In addition, most of these aspects are unpredictable such as the water pressure, the mobility of the animal, and the activities of the underwater, and uneven depths of the underwater surface. Thus, network design and deployment are intricate. In the constrained communication, the currently tethered technology can be used but with additional cost [2].

**The impact of channel structure**: The underwater acoustic channel is looked at as a frequency selective time-varying channel [38]. If the positioning algorithm requires the distance measures, the structure of the channel will decrease the accuracy of the positioning. In [38], the authors show that the range measurement is based on direct LOS signals. However, the direct signal may be lost because of the presence of structures and obstacles in the underwater channel. Thus, on the side of the receiver, the case that only NLOS signals are detected may happen. This means that the NLOS signals may be processed as LOS signals, which lead to the lower accuracy.

**Novel protocols for UWSNs**: In UWSNs, the medium of communication is water but not the air, which is the medium of communication in the terrestrial sensor networks. Thus, the communication protocols of the terrestrial sensor networks do not work efficiently in UWSNs. In addition, the RF signals are mainly considered the short-distance, while the acoustic signals in UWSNs can be used for long-distance communication in water. Thus, a new protocol is needed for UWSNs. Of course, numerous protocols have been put out and we have presented some survey papers of the protocols in UWSNs. However, some improvement and new protocols still need more attention.

**Time synchronization**: As previously mentioned, some schemes do not require the time synchronization, but the other designs need it. In the time synchronization scheme, the time synchronization is an important part to be concerned. The surface nodes are time-synchronized via GPS or DNR, while the underwater nodes cannot be time-synchronized, and their clocks are subject to skew as well as offsets [12]. Some protocols of time synchronization have been proposed in [71,72,73].

**Physical damage to equipment and unreliable information**: In UWSNs, some sensor nodes are located in the water, which may suffer from physical damage. In [2], the authors have pointed out that the algae collection on the camera lens and salt accumulation may lead to the lower effectiveness of sensors. In addition, in the underwater environment, the mobile current, other animals, and many unpredictable factors may lead to dismantling the network topology and node locations. Thus, the information may be unreliable.

**Node deployment and node mobility**: Unlike the node deployment in the WSNs, in the underwater environment, it is more challenging and costly. If deploying the node in the deep sea environment, the researcher may face more challenges. In addition, the node is mobile with the current and other activities [74]. Considering that the speed of the current varies by time and unpredictable, the estimation of a moving node in the localization process may obtain some errors.

**Security and privacy**: Many papers do not take the two aspects into account during the design of the positioning algorithms. However, there is no doubt that they are crucial in UWSNs. In [75], the authors mainly focus on the security attacks on the underwater localization and countermeasures, as well as privacy issues in the underwater localization and countermeasures. A node must reveal certain information to be localized, which may lead to privacy vulnerabilities. Both in the location-related information collection step and the location estimation step the location privacy is discussed, and some techniques to preserve privacy in underwater localization are presented in [75].

The attacks contain DoS attacks, range-based measurement attacks, and range-free measurement attacks, advertising false information and non-cooperation. These techniques for securing underwater localization, which are misbehavior detection, robust location computation, and location verification, will be put forward.

## 6. Conclusions

The localization in UWSNs is similar to the indoor positioning for the WSN where the TOA, AOA, TDOA, and RSS algorithms can be used in UWSNs. However, there are still many differences between the acoustic communications. Because of the unpredictable properties of the underwater environment, more factors that may affect the accuracy of the localization need to be taken into account and more difficulties need to be overcome. In our paper, we first describe the acoustic communication, network architecture, and routing techniques of UWSNs. Different from other survey papers, the positioning algorithms are classified into five aspects based on various criteria, namely, computation algorithm, spatial coverage, range measurement, the state of the nodes and communication between nodes. In each class, the subcategories are contained. Lots of pioneering papers in the field are collected and compared. Moreover, a comprehensive comparison is presented. General challenges and open issues about positioning algorithms based on acoustic communication for UWSNs are also discussed. 

## Figures and Tables

**Table 1:** Comparison of survey papers for the localization algorithms.

Ref.	Taxonomy Method	Reference Number	The Newest Reference	Standard of Comparison	The Related Knowledge
Ours	Computation algorithm, Spatial coverage, Range measurement, The state of the nodes, Communication between nodes	High	2016	Detailed	Medium
[4]	Centralized techniques, Distributed techniques	Medium	2010	Common	Detailed
[10]	Centralized localization techniques, Distributed localization techniques	Low	2011	Detailed	Brief
[11]	Centralized localization techniques, Distributed localization techniques	Low	2010	Detailed	Brief
[12]	Ranging algorithms in UASNs, UASN architecture and localization schemes	Medium	2015	Detailed	Detailed
[13]	Stationary localization algorithms, Mobile localization algorithms, Hybrid localization algorithms	Medium	2012	Medium	Detailed

**Table 2:** Comparison of localization algorithms for using computation algorithms.

Ref.	Computation Algorithm	Coverage	Localization Time	Accuracy	Computational Complexity	Energy Consumption
[24]	Centralized and estimation	Large	Short	Average	Medium	Low
[25]	Centralized and prediction	Small	Short	Average	Low	Low
[6]	Distributed and estimation	Large	Medium	High	Medium	Low
[26]	Distributed and estimation	Large	Medium	High	Medium	Medium
[27]	Distributed and estimation	Small	Long	Depend	Low	Low
[28]	Distributed and estimation	Large	Depend	Depend	Low	High
[29]	Distributed and prediction	Large	Short	Depend	Meidum	Low
[30]	Distributed and prediction	Large	Long	Low	High	Depend

**Table 3:** Comparison of localization algorithms for using spatial coverage.

Ref.	Spatial Coverage	Coverage	Localization Time	Accuracy	Computational Complexity	Energy Consumption
[31]	2D-network and anchor-based	Large	Medium	Low	Medium	Medium
[32]	2D-network and anchor-based	Large	Medium	High	Low	Medium
[33]	3D-network and anchor-free	Large	Medium	Average	Low	Low
[34]	3D-network and anchor-based	Large	Medium	Average	Medium	Medium
[35]	3D-network and anchor-based	Large	Medium	High	Medium	Low
[36]	3D-network and anchor-based	Large	Medium	Average	Medium	Medium
[37]	3D-network and anchor-based	Large	Medium	Average	Medium	Large

**Table 4:** Comparison of localization algorithms for using range measurements.

Ref.	Range Measurement	Coverage	Localization Time	Accuracy	Computational Complexity	Energy Consumption
[39]	Range-free and asynchronous	Large	Short	High	Low	Low
[40]	Range-free and asynchronous	Large	Short	High	Low	Low
[41]	Range-free and asynchronous	Limited	Average	Low	Low	High
[42]	Range-based and synchronous	Large	Medium	Average	Medium	Medium
[43]	Range-based and synchronous	Large	Short	Average	Medium	Low
[44]	Range-based and synchronous	Large	Medium	Low	Medium	Medium
[45]	Range-based and asynchronous	Large	Medium	Average	High	Medium
[46]	Range-based and asynchronous	Large	Medium	Medium	Medium	Medium
[47]	Range-based and asynchronous	Large	Medium	Medium	Low	Low
[48]	Range-based and asynchronous	Large	Short	Depend	Low	Low

**Table 5:** Comparison of localization algorithms for using the state of nodes.

Ref.	The State of the Nodes	Coverage	Localization Time	Accuracy	Computational Complexity	Energy Consumption
[49]	Stationary and active	Large	Long	High	Low	High
[50]	Stationary and active	Large	Depend	Low	Medium	Low
[51]	Stationary and active	Small	Long	Low	Low	High
[52]	Stationary and silent	Small	Short	Low	Low	High
[53]	Stationary and silent	Large	Depend	Average	Medium	Low
[54]	Stationary and silent	Large	Depend	Average	Medium	Medium
[55]	Mobile and active	Large	Long	High	High	High
[56]	Mobile and active	Large	Long	High	High	Low
[57]	Mobile and silent	Large	Short	Average	Low	High
[58]	Mobile and silent	Depend	Depend	Depend	Low	High
[59]	Hybrid and active	Large	Depend	Average	Low	Low
[60]	Hybrid and silent	Large	Short	Low	Low	Low
[61]	Hybrid and silent	Large	Depend	Depend	Low	High

**Table 6:** Comparison of localization algorithms for using communication between nodes.

Ref.	Communication between Nodes	Coverage	Localization Time	Accuracy	Computational Complexity	Energy Consumption
[62]	Single-stage	Medium	Average	High	Low	High
[64]	Mutil-stage	Large	Long	Low	High	High
[65]	Mutil-stage	Small	Depend	Average	Medium	Medium

**Table 7:** Comparison of the localization algorithms.

Ref.	C or D	E or P	2D or 3D	Af or Ab	Rf or Rb	Sy or As	Sa or M or H	A or Si	Ss or Ms
[6]	√	√	√	√	√	Not	Not	√	√
[17,24]	√	√	√	√	√	√	√	√	√
[25]	√	√	√	√	√	√	√	√	√
[26]	√	√	√	√	√	Not	√	√	√
[27]	√	√	√	√	√	√	√	√	√
[28]	√	√	√	√	√	√	√	√	√
[29]	√	√	√	√	√	Not	√	√	√
[30,66]	√	√	√	√	√	√	√	√	√
[31,32]	√	√	√	√	√	√	√	Not	√
[33]	√	√	√	√	√	Not	√	√	√
[67]	√	√	√	√	Not	Not	√	√	√
[34]	√	√	√	√	√	√	Not	Not	√
[35]	√	√	√	√	√	√	√	√	√
[36]	√	√	√	√	√	Not	Not	√	√
[37]	√	√	√	√	√	√	√	√	√
[39]	√	√	√	√	√	√	√	√	√
[40]	√	√	√	√	√	√	√	√	√
[41]	√	√	√	√	√	√	√	√	√
[68,69]	√	√	√	√	√	√	√	√	√
[70]	√	√	√	√	√	√	√	Not	√
[42]	√	√	√	√	√	√	Not	√	√
[43]	√	√	√	√	√	√	√	√	√
[44]	√	√	√	√	√	√	Not	√	√
[54]	√	√	√	√	√	√	√	√	√
[45]	√	√	√	√	√	√	√	√	√
[46]	√	√	√	√	√	√	√	√	√
[47]	√	√	√	√	√	√	Not	√	Not
[48]	√	√	√	√	√	√	√	√	√
[49]	√	√	√	√	√	√	√	√	√
[50]	√	√	√	√	√	√	√	√	√
[51]	√	√	√	√	Not	Not	√	√	√
[52]	√	√	√	√	Not	Not	√	√	√
[63]	√	√	√	√	√	√	√	√	√
[53]	√	√	√	√	√	√	√	√	√
[55,56]	√	√	√	√	√	√	√	√	√
[57]	√	√	√	√	√	√	√	√	√
[58]	√	√	√	√	√	√	√	√	√
[59]	√	√	√	√	√	√	√	√	√
[60]	√	√	√	√	√	√	√	√	√
[61]	√	√	√	√	√	√	√	√	√
[62]	√	√	√	√	√	√	√	√	√
[64]	√	√	√	√	√	√	√	√	√
[65]	√	√	√	√	√	√	√	√	√

## References

[1] Kumar R., Singh N. (2014). A survey on data aggregation and clustering schemes in underwater sensor networks. Int. J. Grid Distrib. Comput..

[2] Felemban E., Shaikh F.K., Qureshi U.M., Sheikh A.A., Qaisar S.B. (2015). Underwater sensor network applications: A comprehensive survey. Int. J. Distrib. Sens. Netw..

[3] Heidemann J., Ye W., Wills J., Syed A., Li Y. Research challenges and applications for underwater sensor networking. Proceedings of the IEEE Wireless Communications and Networking Conference.

[4] Erol-Kantarci M., Mouftah H.T., Oktug S. (2011). A survey of architectures and localization techniques for underwater acoustic sensor networks. IEEE Commun. Surv. Tutor..

[5] Ren Y., Yu N., Wang X., Wan J. (2013). Set-membership localization algorithm based on adaptive error bounds for large-scale underwater wireless sensor networks. Electron. Lett..

[6] Allotta B., Bartolini F., Caiti A., Costanzi R., Corato F.D., Fenucci D., Gelli J., Guerrini P., Monni N., Natalini M. (2015). Typhoon at commsNet13: Experimental experience on AUV navigation and localization. Annu. Rev. Control.

[7] Zenia N.Z., Aseeri M., Ahmed M.R., Chowdhury Z.I., Shamim Kaiser M. (2016). Energy-efficiency and Reliability in MAC and Routing Protocols for Underwater Wireless Sensor Network. J. Netw. Comput. Appl..

[8] Lloret J. (2013). Underwater sensor nodes and networks. Sensors.

[9] Heidemann J., Stojanovic M., Zorzi M. (2011). Underwater sensor networks: Applications, advances and challenges. Philos. Trans. R. Soc. A Math. Phys. Eng. Sci..

[10] Erol-Kantarci M., Mouftah H.T., Oktug S. (2010). Localization techniques for underwater acoustic sensor networks. IEEE Commun. Mag..

[11] Nageswararao K., Prasan U.D. (2012). A survey on underwater sensor networks localization techniques. Int. J. Eng. Res. Dev..

[12] Qu F.Z., Wang S.Y., Wu Z.H., Liu Z.B. (2016). A survey of ranging algorithms and localization schemes in underwater acoustic sensor network. China Commun..

[13] Han G., Jiang J., Shu L., Xu Y., Wang F. (2012). Localization algorithms of underwater wireless sensor networks: A survey. Sensors.

[14] Ayaz M., Baig I., Abdullah A., Faye I. (2011). A survey on routing techniques in underwater wireless sensor networks. J. Netw. Comput. Appl..

[15] Li N., Martinez J.F., Chaus J.M.M., Eckert M. (2016). A survey on underwater acoustic sensor network routing protocols. Sensors.

[16] Chen K., Ma M., Cheng E., Yuan F., Su W. (2014). A survey on MAC protocols for underwater wireless sensor networks. IEEE Commun. Surv. Tutor..

[17] Moradi M. (2013). A Reverse Localization Scheme for Underwater Acoustic Sensor Networks.

[18] Li D., Du J., Liu L. A data routing algorithm based on Markov model in underwater wireless sensor networks. Proceedings of the 2016 IEEE International Conference on Ubiquitous Wireless Broadband (ICUWB).

[19] Khan G., Gola K.K., Ali W. Energy Efficient Routing Algorithm for UWSNs—A Clustering Approach. Proceedings of the 2015 Second International Conference on Advances in Computing and Communication Engineering.

[20] Pervaiz K., Wahid A., Sajid M., Khizar M., Khan Z.A., Qasim U., Javaid N. DEAC: Depth and Energy Aware Cooperative Routing Protocol for Underwater Wireless Sensor Networks. Proceedings of the 2016 10th International Conference on Complex, Intelligent, and Software Intensive Systems (CISIS).

[21] Khan T., Ahmad S., Babar M.I. Role of Anycasting in Threshold-Optimized Depth-Based Routing Protocol for Underwater Wireless Networks. Proceedings of the 2016 International Conference on Computational Science and Computational Intelligence (CSCI).

[22] Javaid N., Cheema S., Akbar M., Alrajeh N., Alabed M.S., Guizani N. (2017). Balanced Energy Consumption Based Adaptive Routing for IoT Enabling Underwater WSNs. IEEE Access.

[23] Hsu C.C., Liu H.H., Gomez J.L.G., Chou C.F. (2015). Delay-Sensitive Opportunistic Routing for Underwater Sensor Networks. IEEE Sens. J..

[24] Moradi M., Rezazadeh J., Ismail A.S. (2012). A Reverse Localization Scheme for underwater acoustic sensor networks. Sensors.

[25] Mirza D., Schurgers C. Collaborative localization for fleets of underwater drifters. Proceedings of the OCEANS.

[26] Liu L., Wu J., Zhu Z. (2015). Multihops fitting approach for node localization in underwater wireless sensor networks. Int. J. Distrib. Sens. Netw..

[27] Luo H., Dong W., Hong F. (2010). LDB: Localization with directional beacons for sparse 3D underwater acoustic sensor networks. J. Netw..

[28] Lee S., Kim K. (2012). Localization with a mobile beacon in underwater acoustic sensor networks. Sensors.

[29] Bhuvaneswari P.T., Karthikeyan S., Jeeva B., Prasath M.A. An efficient mobility based localization in underwater sensor networks. Proceedings of the 2012 Fourth International Conference on Computational Intelligence and Communication Networks.

[30] Zhou Z., Peng Z., Cui H.J., Shi Z., Bagtzoglou A.C. (2011). Scalable localization with mobility prediction for underwater sensor networks. IEEE Trans. Mob. Comput..

[31] Diamant R., Wolff L.M., Lampe L. (2015). Location tracking of ocean-current-related underwater drifting nodes using Doppler shift measurements. IEEE J. Ocean. Eng..

[32] Dong B., Mahdy A.M. (2010). Underwater wireless sensor networks: efficient localization schemes using semi-definite programming. Int. J. Adv. Netw. Serv..

[33] Guo Y., Liu Y. (2013). Localization for anchor-free underwater sensor networks. Comput. Electr. Eng..

[34] Ren Y., Zhong J., Huang J., Song Y., Xin X., Yu N., Feng R. (2014). Orthogonal Regression Based Multihop localization algorithm for large-scale underwater wireless sensor networks. Int. J. Distrib. Sens. Netw..

[35] Uddin M.Y.S. Low-overhead range-based 3D localization technique for underwater sensor networks. Proceedings of the 2016 IEEE International Conference on Communications (ICC).

[36] SenLin Z., Qiang Z., MeiQin L., Zhen F. (2013). A top-down positioning scheme for underwater wireless sensor networks. Sci. China Inf. Sci..

[37] Liu Z., Gao H., Wang W., Chang S., Chen J. (2015). Color filtering localization for three-dimensional underwater acoustic sensor networks. Sensors.

[38] Tan H.P., Diamant R., Seah W.K., Waldmeyer M. (2011). A survey of techniques and challenges in underwater localization. Ocean Eng..

[39] Zandi R., Kamarei M., Amiri H., Yaghoubi F. (2015). Underwater sensor network positioning using an AUV Moving on a Random Waypoint Path. IETE J. Res..

[40] Zandi R., Kamarei M., Amiri H. (2016). Distributed estimation of sensors position in underwater wireless sensor network. Int. J. Electron..

[41] Chandrasekhar V., Seah W. An area localization scheme for underwater sensor networks. Proceedings of the OCEANS 2006—Asia Pacific.

[42] Wu Z., Li X. (2015). An improved underwater acoustic network localization algorithm. China Commun..

[43] Zhu S., Jin N., Wang L., Zheng X., Yang S., Zhu M. A novel dual-hydrophone localization method in underwater sensor networks. Proceedings of the 2016 IEEE/OES China Ocean Acoustics (COA).

[44] Diamant R., Lampe L. (2013). Underwater localization with time-synchronization and propagation speed uncertainties. IEEE Trans. Mob. Comput..

[45] Carroll P., Mahmood K., Zhou S., Zhou H., Xu X., Cui J.H. (2014). On-demand asynchronous localization for underwater sensor networks. IEEE Trans. Signal Process..

[46] Emokpae L.E., DiBenedetto S., Potteiger B., Younis M. (2014). UREAL: Underwater reflection-enabled acoustic-based localization. IEEE Sens. J..

[47] Mirza1 M.A., Shakir M.Z., Alouini3 M.S. (2013). A scalable global positioning system-free localization scheme for underwater wireless sensor networks. EURASIP J. Wirel. Commun. Netw..

[48] Isbitiren G., Akan O.B. (2011). Three-dimensional underwater target tracking with acoustic sensor networks. IEEE Trans. Veh. Technol..

[49] Liu B., Chen H., Zhong Z., Poor H.V. (2010). Asymmetrical round trip based synchronization-free localization in large-scale underwater sensor networks. IEEE Trans. Wirel. Commun..

[50] Zhang S., Li D., Li L., Liao Z. (2014). Loop assisted synchronization-free localization for underwater acoustic sensor networks. Int. J. Distrib. Sens. Netw..

[51] Watfa M.K., Nsouli T., Al-Ayache M., Ayyash O. Reactive localization in underwater wireless sensor networks. Proceedings of the 2010 Second International Conference on Computer and Network Technology (ICCNT).

[52] Teymorian A.Y., Cheng W., Ma L., Cheng X., Lu X., Lu Z. (2009). 3D underwater sensor network localization. IEEE Trans. Mob. Comput..

[53] Ren Y., Yu N., Guo X., Wan J. Cube-scan-based three dimensional localization for large-scale underwater wireless sensor networks. Proceedings of the 2012 IEEE International Systems Conference (SysCon).

[54] Beniwal M., Singh R., Sangwan A. (2016). A localization scheme for underwater sensor networks Without Time Synchronization. Wirel. Pers. Commun..

[55] Kim S., Yoo Y. (2014). SLSMP: Time synchronization and localization using seawater movement pattern in underwater wireless networks. Int. J. Distrib. Sens. Netw..

[56] Liu J., Wang Z., Cui J.H., Zhou S., Yang B. (2016). A joint time synchronization and localization design for mobile underwater sensor networks. IEEE Trans. Mob. Comput..

[57] Erol M., Vieira L.F.M., Caruso A., Paparella F., Gerla M., Oktug S. Multi stage underwater sensor localization using mobile beacons. Proceedings of the Second International Conference on Sensor Technologies and Applications 2008 (SENSORCOMM ’08).

[58] Erol M., Vieira L.F.M., Gerla M. AUV-aided localization for underwater sensor networks. Proceedings of the International Conference on Wireless Algorithms, Systems and Applications 2007 (VASA 2007).

[59] Isik M.T., Akan O.B. (2009). A three dimensional localization algorithm for underwater acoustic sensor networks. IEEE Trans. Wirel. Commun..

[60] Callmer J., Skoglund M., Gustafsson F. (2010). Silent localization of underwater sensors using magnetometers. EURASIP J. Adv. Signal Process..

[61] Lee S., Kim K. Localization with a Mobile Beacon in Underwater Sensor Networks. Proceedings of the 2010 IEEE/IFIP 8th International Conference on Embedded and Ubiquitous Computing (EUC).

[62] Bian T., Venkatesan R., Li C. Design and evaluation of a new localization scheme for underwater acoustic sensor networks. Proceedings of the Global Telecommunications Conference 2009 (GLOBECOM 2009).

[63] Tan H.P., Gabor A.F., Eu Z.A., Seah W.K.G. A wide coverage positioning system (WPS) for underwater localization. Proceedings of the 2010 IEEE International Conference on Communications (ICC).

[64] Zhou Z., Cui J.H., Zhou S. (2007). Localization for large-scale underwater sensor networks. NETWORKING 2007. Ad Hoc and Sensor Networks, Wireless Networks, Next Generation Internet.

[65] Erol M., Oktug S. A localization and routing framework for mobile underwater sensor networks. Proceedings of the INFOCOM Workshops 2008.

[66] Zhou Z., Cui J.H., Bagtzoglou A. Scalable localization with mobility prediction for underwater sensor networks. Proceedings of the 2008 IEEE INFOCOM—The 27th Conference on Computer Communications.

[67] Othman A.K., Adams A.E., Tsimenidis C.C. Node discovery protocol and localization for distributed underwater acoustic networks. Proceedings of the International Conference on Internet and Web Applications and Services/Advanced International Conference on Telecommunications (AICT-ICIW’06).

[68] Yi Z., Juna G.B., Kai C., Jianboa C., Haibinga G. (2009). An range-free localization scheme for large scale underwater wireless sensor networks. J. Shanghai Jiaotong Univ. Sci..

[69] Zhou Y., He J., Chen K., Chen J., Liang A. An area localization scheme for large scale underwater wireless sensor networks. Proceedings of the CMC ’09.

[70] Hea T., Huang C., Blum B.M., Stankovic J.A., Abdelzaher T. Range-free localization schemes for large scale sensor networks. Proceedings of the 9th Annual International Conference on Mobile Computing and Networking (MobiCom ’03).

[71] Chirdchoo N., Soh W.S., Chua K.C. Mu-sync: A time synchronization protocol for underwater mobile networks. Proceedings of the Third ACM International Workshop on Underwater Networks.

[72] Syed A.A., Heidemann J. Time synchronization for high latency acoustic networks. Proceedings of the 25th IEEE International Conference on Computer Communications (INFOCOM 2006).

[73] Chen H.C., Xu W. Simplified time synchronization for underwater acoustic sensor networks with high propagation latency. Proceedings of the OCEANS 2014-TAIPEI.

[74] Beniwal M., Singh R. (2014). Localization techniques and their challenges in underwater wireless sensor networks. Int. J. Comput. Sci. Inf. Technol..

[75] Li H., He Y.H., Cheng X.Z., Zhu H.S., Sun L.M. (2015). Security and privacy in localization for underwater sensor networks. IEEE Commun. Mag..

